# The Legal Responsibility of Physicians

**Published:** 1882-05-20

**Authors:** 


### THE LEGAL RESPONSIBILITY OF PHYSICIANS.

There are very few medical gentlemen who thoroughly realize the importance of some knowledge of the legal responsibilities they may incur in the daily routine of the profession. The British Medical Journal, in a recent issue, relates several striking instances of this unsuspected danger. In one case a woman, who was arrested on suspicion of having concealed the birth of a child, was examined by the Police Surgeon, to ascertain whether she had recently been confined or not, although she had previously confessed to the birth, and remarked that "there was no use in examining her." She subsequently brought an action for damages against this physician, on the ground of assault; the presiding Justice charged the jury in these words: "Before the examination took place she admitted having had a child; the jury must ask themselves, if that admission was made, why examine further; there was no legal authority whatever to examine this girl." After a short absence the jury returned a verdict of one hundred and twenty-five dollars damages for the plaintiff.

Some years ago one of our most prominent surgeons in this city was called upon to attend a fracture of the femur. The bone was set and the limb placed in proper position, while careful instructions were given for future management, and absolute rest enjoined. The latter injunction was disobeyed, erysipelas supervened, and shortening of the leg was the result. A suit for damages followed, and while the surgeon was successful, yet he was forced to much undeserved expense and anoyance. Many such cases are reported and could be cited to prove our position. We must ever remember that there are many unprincipled persons in the world, and also anxious, eager, vulture-like lawyers, ever ready to whisper into the ear of a willing client, "Sue him for damages; he has money; he has treated you badly, make him pay for it;" and there are many ready and willing to listen to this evil advice. Some of the English courts hold that whenever a physician makes an examination of a woman without having her clearly expressed consent, he is guilty of an *assault*. She may not resist, they claim, because there may be, to her knowledge, some collateral circumstances capable of forcing her acquiescence if she does refuse; though these fears may not be well grounded, yet if they seem to her sufficient to make resistance, to her mind, useless, then such an unwilling examination constitutes assault. They even decided, as far back as 1824, "That if a medical man unnecessarily strip a female patient, under the pretense that he cannot otherwise judge of her illness, it is an assault, if he himself takes off her clothes.

These decisions are all eminently just and proper. Morality is one of the greatest foundation stones of social prosperity, and must be most zealously guarded. The purity and privacy of the female must be rigidly maintained, if morality is to be encouraged, and this barrier which must ever surround the modesty of woman can only be passed when the absolute necessities of deranged health imperatively demand it, and then exposure must only be carried to such degree as is indispensably necessary. These decisions are based on the unnecessary exposure of the female, and are, therefore, just.

Since there are many women who are ready to be induced to bring suit against physicians on the slightest provocation, or even, indeed, without any just cause at all, it, therefore, will be a wise precaution to bear ever in mind the fact that it has been decided that examination without express consent constitutes assault.

To avoid this trouble, in which many have and many more will innocently find themselves, unless they heed this caution, it should be made a rule to previously obtain full and free consent from a woman, before a witness or witnesses, ere you undertake an examination, and to surround yourself with the additional safeguard of a witness during the operation. The relations sometimes existing between physician and patient are of an extremely delicate nature, and unless carefully and cautiously conducted, may, in certain instances, redound greatly to the detriment of the physician.
—*Med. and Surg. Rep.*

---

**Incontinence of Urine.**—For incontinence of urine in children Dr. Janaway (New York Med. Record) recommends a combination of ergot, belladonna, and iodide of iron. He says that this prescription is more useful in this affection than any combination of drugs known.—*Ex.*

